# Pharmacological and Non-Pharmacological Interventions in Diabetes Mellitus: Effects on Epicardial Adipose Tissue

**DOI:** 10.3390/ijms26199271

**Published:** 2025-09-23

**Authors:** Krzysztof Kuleta, Kamil Krauz, Jakub Żmuda, Karol Momot, Maciej Zarębiński, Izabela Poprawa, Małgorzata Wojciechowska

**Affiliations:** 1 Chair and Department of Experimental and Clinical Physiology, Laboratory of Centre for Preclinical Research, Medical University of Warsaw, Banacha 1b, 02-097 Warsaw, Poland; krzysiekkuleta01@gmail.com (K.K.); kamil.krauz@wum.edu.pl (K.K.); zmudajakub875@gmail.com (J.Ż.); karol.momot@wum.edu.pl (K.M.); 2 Doctoral School, Medical University of Warsaw, 02-097 Warsaw, Poland; 3 Department of Invasive Cardiology, Independent Public Specialist Western Hospital John Paul II, Lazarski University, Daleka 11, 05-825 Grodzisk Mazowiecki, Poland; maciej@zarebinski.pl (M.Z.); izapoprawa@gmail.com (I.P.)

**Keywords:** epicardial adipose tissue, diabetes mellitus, anti-diabetic treatment

## Abstract

Diabetes mellitus (DM) has emerged as a significant issue for both individual patients and global health. Elevated blood glucose levels lead to complications affecting the cardiovascular system. Due to this fact and the growing population of patients with DM, it is crucial to broaden the knowledge concerning DM pathogenesis, allowing for the prevention and alleviation of organ-specific complications. Nowadays, pharmacological and non-pharmacological approaches are implicated in DM management. Epicardial adipose tissue (EAT) was indicated to modulate the impact of diabetes mellitus on the heart. Emerging evidence indicates that antidiabetic drugs can significantly influence EAT, often independently of their glucose-lowering effects, suggesting additional cardiometabolic benefits. However, not all drug classes, and even agents within the same class, exhibit identical effects on EAT, highlighting that some therapies may be preferred over others for cardiovascular benefit. Lifestyle interventions, commonly recommended to patients with DM, might also target epicardial fat. This article extensively reviews the impact of current DM treatment on EAT and depicts potential mechanisms. It also aims to identify gaps in knowledge and potential future directions. Insights from this work may guide future research and therapeutic strategies aimed at reducing cardiovascular risk in patients with DM.

## 1. Introduction

Diabetes mellitus (DM) can be defined as a group of metabolic disorders of carbohydrate metabolism involving both underutilization of glucose as an energy source and its overproduction due to inappropriate gluconeogenesis and glycogenolysis, which results in hyperglycemia [1]. It constitutes a significant public health concern. Its prevalence has rapidly grown over recent decades and reached approximately 828 million in 2022. Type 2 DM (T2DM), traditionally linked with insulin resistance and dysfunction of β-cells, accounts for about 95% of cases [2]. Among multiple environmental factors, reduced physical activity, improper dietary habits leading to obesity, and psychological stress contribute to its development [3]. In addition, genetic predisposition plays a significant role in T2DM susceptibility [4]. Type 1 DM is an autoimmune disease and results from immune-system-mediated damage to pancreatic β-cells [5].

Recently, epicardial adipose tissue (EAT) has emerged as a vital player in the pathogenesis of multiple cardiovascular pathologies [6]. Also, DM is a significant risk factor for their development [7]. Therefore, EAT may mediate the pathogenic effect of DM on the myocardium and coronary arteries, as EAT is not simply a passive fat depot but an active endocrine organ linking metabolic disturbances to cardiovascular complications [8,9,10]. Consistently, increased EAT volume has been associated with coronary artery disease among humans [11,12]. There is growing body of evidence documenting the association between pathological expansion of EAT and onset, as well as severity of atrial fibrillation (AF) [13,14]. Several mechanisms, including atrial myocardium fat infiltration, release of pro-inflammatory cytokines, and increased oxidative stress, have been suggested to explain how EAT contributes to AF [13,15,16]. Moreover, greater volume and more positive attenuation values of EAT surrounding the left atrium are associated with AF recurrence following catheter ablation [17,18].

Efficient DM treatment lowers blood glucose levels and is essential for preventing or delaying complications. It involves both pharmacological interventions and lifestyle modifications, with the latter playing a particularly important role in T2DM [19]. Among pharmacological methods, we discuss following medications used in DM management: glucagon-like peptide-1 receptor agonists, sodium-glucose cotransporter-2 inhibitors, metformin, thiazolidinediones, dipeptidyl peptidase-4 inhibitors, insulin, and sulfonylureas. In the “Non-pharmacological interventions” section we included physical exercise, diet, bariatric surgery, and a description of studies involving combinations of these methods. However, the mechanisms linking DM treatment to EAT modulation remain insufficiently understood. A deeper understanding of these relationships may lead to a reduction in cardiovascular complications in patients with DM.

This review primarily aims to discuss the changes in EAT due to both pharmacological and non-pharmacological management of DM. It also provides a brief overview of the general characteristics of EAT and its role in DM.

## 2. EAT Characteristics

### 2.1. Anatomy

EAT is a component of the visceral adipose tissue (VAT) located between the myocardium and the visceral layer of the pericardium. It is important to differentiate epicardial fat from pericardial fat, which is situated between the visceral and parietal layers of the pericardium [20]. EAT is predominantly found in the atrioventricular and interventricular grooves. Pericoronary EAT, which surrounds the coronary arteries, and myocardial EAT, which is located directly over the myocardium, can be differentiated within EAT [6,21]. EAT constitutes approximately 20% of the total heart mass. Nevertheless, its volume and distribution might vary based on factors such as sex, genetic profile, and environment [21,22]. No anatomical obstacle, such as muscle fascia, separates EAT from the myocardium, allowing epicardial fat to infiltrate the myocardial tissue [23]. Also, both EAT and myocardium share the same blood supply from the coronary arteries [21,24], while pericardial fat is supplied by branches of the aorta [20]. Therefore, their anatomical adjacency and shared microcirculation suggest the existence of direct crosstalk between epicardial fat and the myocardium. EAT originates from splanchnopleuric mesoderm, unlike pericardial fat, which originates from thoracic mesenchyme [25]. Microscopically, adipocytes present in epicardial fat are generally smaller than those located in other visceral fat depots, which may prevent large lipid storage [26]. Epicardial adipocytes, together with numerous other types of cells such as immune cells, nerve cells, stromal cells, and vascular cells, form a unique microenvironment that might significantly interfere with the myocardium [27].

### 2.2. White or Brown?

EAT is generally considered white adipose tissue. However, it also exhibits brown fat-like and beige fat-like features [28,29]. It has been demonstrated that uncoupling protein-1 (UCP-1), a specific marker of brown adipose tissue (BAT), is highly expressed in epicardial fat. UCP-1 uncouples oxidative phosphorylation in mitochondria, redirecting energy to thermogenesis instead of ATP synthesis [30]. Thus, it indicates that EAT is involved in heat production. The presence of brown adipocytes interspersed among white adipocytes is not exclusive to epicardial fat. Some authors defined these cells as BRITE (brown-in-white) [31,32,33]. Nevertheless, the percentage of brown adipocytes in EAT is significantly higher than in other fat depots. Sacks et al. demonstrated that expression of UCP-1 among patients undergoing heart surgery was approximately 90 times greater in EAT than in leg subcutaneous adipose tissue [29]. It has been shown that diabetes impairs glucose uptake and causes whitening of BAT in diabetic rat models [34]. Among older individuals, the proportion of brown adipocytes decreases, while the number of white adipocytes increases [35]. This could contribute to impairment of heart function in the elderly. Moreover, in chronic ischemia, such as in advanced coronary heart disease (CAD), the activity of BAT in EAT also decreases [36]. This indicates that the content and activity of BAT in EAT is not constant. The browning of adipose tissue continues to draw scientific attention due to its possible contribution to the treatment and prevention of T2DM and other prevalent metabolic disorders.

### 2.3. Role of EAT

Under physiological conditions, epicardial fat exerts a protective effect on the heart, contributing to the myocardial homeostasis [37]. For instance, it exhibits its brown adipose-like characteristics by contributing to thermoregulation and securing the heart from hypothermia [29]. Moreover, it may serve as one of the primary energy sources for the heart, additionally protecting it from high free acid levels and local lipotoxicity [38]. Beyond its thermoregulatory and metabolic roles, EAT further supports the myocardium by producing cardioprotective adipokines such as adiponectin and adrenomedullin, both presenting anti-inflammatory and anti-atherogenic features [39,40]. By surrounding the heart and its vessels, EAT provides mechanical protection and a site for autonomic ganglia which innervate the myocardium [37]. Nevertheless, EAT might be deeply harmful under pathological conditions, which will be discussed further.

### 2.4. EAT Assessment

EAT can be quantitatively and, depending on the imaging modality, also qualitatively assessed by imaging techniques such as echocardiography, computed tomography, magnetic resonance imaging, and 18F-fluorodeoxyglucose positron emission tomography-computed tomography. Assessed parameters of EAT may involve thickness, volume, and density [6]. The latter, also referred to as EAT attenuation, can be measured using computed tomography. It is a value expressed in Hounsfield units and can possibly reflect the hypertrophy and/or hyperplasia of adipocytes as well as inflammatory and fibrotic changes in EAT [18,41,42]. EAT volume can be measured. Assessment of EAT with echocardiography is affordable, non-invasive, and widely available which makes it possible to use in clinical practice. However, it only allows for measurement of EAT thickness. Another significant limitation is intraoperator variability [6].

## 3. How Does EAT Change in Diabetes?

As previously described, among healthy individuals, EAT plays a cardioprotective role. Nevertheless, this equilibrium may be disturbed under the influence of pathological conditions, such as DM, causing the change in the structure and functions of EAT.

Several studies have been performed to assess EAT expansion among patients with DM. It has been demonstrated that among patients with DM, compared to non-diabetic individuals, both EAT volume (166.1 ± 60.6 cm^3^ vs. 123.4 ± 41.8 cm^3^) [43] and thickness (6.4 ± 1.7 mm vs. 3.3 ± 1.1 mm) [44] are significantly greater. EAT expansion is driven by factors such as insulin resistance and metabolic changes occurring in diabetes. In one of the studies conducted in obese children, EAT thickness positively correlated with insulin resistance and was also an independent predictor of insulin resistance [45]. Moreover, patients with DM may have shifted values of EAT attenuation towards more negative values. Lower attenuation values can be explained by an increase in lipid-laden adipocyte population due to calorie excess [46]. However, inflammation should rather cause a shift in EAT attenuation towards more positive values [47].

EAT in DM may mediate deleterious effects on the myocardium. Under pathological conditions such as diabetes, EAT may exhibit a pro-inflammatory phenotype and become a source of inflammatory and pro-fibrotic cytokines, including tumor necrosis factor alpha (TNF-α), interleukin-1β, and interleukin 6 (IL-6) [48]. Among patients with coronary artery disease (CAD), T2DM has been associated with decreased expression of proliferator-activated receptor gamma coactivator 1-alpha (PGC1α) and UCP1 mRNA. These changes could suggest the loss of beneficial brown-like fat features [49]. Also, signaling promoted by advanced glycation end products in EAT may stimulate oxidative stress and endothelial damage and, therefore, contributing to development and progression of coronary atherosclerosis in patients with DM [6]. Taken together, these factors may exert pro-fibrotic and pro-atherogenic properties as well as contribute to the development of diabetic cardiomyopathy. However, these data have been extensively reviewed so far, and they are beyond the scope of this article [8,50].

## 4. Pharmacological Interventions

The intricate relationship between diabetes and cardiovascular disease is well recognized, with both conditions frequently coexisting and driving the progression of metabolic dysfunction [51,52]. One of the key links between these conditions may be the involvement of EAT. This is why, in recent years, there has been growing interest in the influence of anti-diabetic medications on EAT—the modifiable risk factor of cardiovascular disease [53]. The impact of some of them has already been investigated. Summary of the main included studies is presented in Table 1.

### 4.1. Glucagon-like Peptide-1 Receptor Agonists

A class of drugs that has attracted significant attention is glucagon-like peptide-1 receptor (GLP-1) agonists. They are subcutaneously injectable medications indicated for the treatment of T2DM. By mimicking endogenous GLP-1 activity, they modulate the incretin pathway. Apart from glucose level control, GLP-1 analogs exhibit pleiotropic impact, including cardiovascular benefits [69]. The exact mechanism of their influence on the cardiovascular system remains not fully understood.

EAT expresses the GLP-1 receptor, which makes it a possible target for GLP-1 agonist therapy [70]. Moreover, the expression of the GLP-1 receptor in EAT is more abundant than in subcutaneous adipose tissue [71]. Thus, this group of drugs may directly affect EAT, independent of their glucose-lowering properties. GLP-1 receptors have also been found directly on cardiomyocytes, which might contribute to GLP-1 analogs’ pleiotropic effect [72].

In an ultrasonographic study, Morano et al. evaluated the effect of GLP-1 analogs—exenatide and liraglutide—on fat distribution among individuals with diabetes [54]. The authors reported that only a 3-month treatment with GLP-1 analogs induced overall weight loss, with a more pronounced reduction in EAT compared to other fat depots. No significant differences were observed between the exenatide and liraglutide treatment groups. Similarly, Iacobellis et al. demonstrated that the addition of liraglutide to metformin therapy in patients with DM resulted in an approximately 30% reduction in EAT thickness after only 3 months of treatment, whereas no significant reduction was observed in the group receiving metformin alone [55]. The same group further investigated the effect of other GLP-1 analogs—semaglutide and dulaglutide. After 3 months of treatment, patients with T2DM showed smaller than in previous study, yet impressive, a 20% decrease in EAT thickness in both groups [56]. The reduction in epicardial fat thickness was found to be dose-dependent, showing significant difference in groups receiving higher doses of semaglutide (1 mg) and dulaglutide (1.5 mg), respectively. These findings might suggest the class effect of GLP-1 receptor agonists on EAT.

GLP-1 receptor activation is associated with fatty acid beta-oxidation and EAT differentiation into BAT. The expression of GLP-1 receptors has been shown to correlate with the expression of genes such as *GATA3*, *FOXC2*, *PPARGC1A*, *SRC,* and *UCP1*, which are involved in BAT activation and white-to-brown fat differentiation [73]. Metabolically active BAT not only contributes to thermogenesis but also plays a key role in triglyceride clearance and insulin sensitivity [74], providing a protective mechanism against excessive fat accumulation [75]. *WNT1*, a gene which encodes for factors involved in inhibition of adipogenesis, has also been found to correlate with GLP-1 receptor expression [73].

GLP-1 analog treatment is also associated with a significant decrease in the levels of low-density lipoprotein (LDL) and triglycerides (TG) in EAT, which are characteristic changes in the improvement of insulin resistance [76]. Exenatide, a GLP-1 receptor agonist, has also been shown to improve aortic pulse wave velocity (PWV) [77], a parameter of aortic stiffness as well as a marker of vascular aging and a prognostic indicator for cardiovascular disease [78]. Furthermore, liraglutide treatment has been demonstrated to improve left ventricular (LV) stiffness, reduce diastolic dysfunction, and reduce mortality among patients with heart failure with preserved ejection fraction (HFpEF) [79]. Preoperative administration of liraglutide also induces better intraoperative glucose control, as demonstrated in coronary artery by-pass grafting (CABG) surgery [80].

Tirzepatide (TZT) is a novel drug with a dual agonist action on GLP-1 and glucose-dependent insulinotropic polypeptide (GIP) receptors, both of which belong to the incretin system [81]. Therefore, some authors refer to it as a “twincretin” [82,83]. Expression of not only GLP-1 receptors but also GIP receptors in epicardial fat makes it a possible target for TZT therapy, which could possibly have beneficial cardiovascular effect beyond glucose control and weight loss [70]. TZT has already been found to exhibit cardioprotective properties. By inhibiting the TLR4/NF-kB/NLRP3 pathway, TZT attenuates lipopolysaccharide-induced left ventricular remodeling and dysfunction in mice [84]. An in vitro study performed by Taktaz et al. demonstrated that TZT protects human cardiomyocytes from diabetes-related damage by positive modulation of cell death, fibrosis, and hypertrophy [85]. For instance, TZT has downregulated expression of the pro-apoptotic protein BAX, while upregulating the anti-apoptotic BCL2. It has also decreased levels of fibrosis markers such as TGF-β or MMP9. Among humans, TZT therapy has been shown to reduce the risk of major adverse cardiovascular events [85]. Kramer et al. have found TZT to significantly reduce LV mass among obese patients with HFpEF, which is suspected to reduce HF events [57]. In the same study, TZT has been shown to significantly reduce pericardial adipose tissue volume but not EAT volume. However, TZT remains a novel drug and evidence regarding its effect on EAT is very limited. Therefore, its potential to modulate EAT should not be disregarded.

T2DM is associated with a chronic low-grade inflammatory state, with elevated levels of circulating inflammatory cytokines [86,87]. Similarly, oxidative stress biomarkers have been found to be increased among individuals with T2DM [88]. Thus, anti-inflammatory and antioxidant effects of GLP-1 receptor analogs might contribute to their beneficial effect on EAT. They have been proven to significantly reduce serum levels of inflammatory biomarkers, such as C-reactive protein (CRP) and tumor necrosis factor-alpha (TNFα), as well as malondialdehyde (MDA)—an oxidative stress biomarker [89].

### 4.2. Sodium-Glucose Cotransporter-2 Inhibitors

Sodium-glucose cotransporter-2 (SGLT-2) inhibitors are a first-line therapy for patients with T2DM and HF [90]. They are hypoglycemic agents that lower blood glucose levels by inhibiting glucose reabsorption in the proximal tubule of the kidney. It allows the excess glucose to be eliminated through urine. SGLT-2 inhibitors exhibit multiple metabolic beneficial effects, including glycemic control, weight loss, and cardiovascular and renal protection. They significantly reduce the incidence of cardiovascular events (CV) or HF, but the mechanism is still discussed [91]. SGLT-2 inhibitors’ cardioprotective effect has been shown to occur irrespective of their glucose-lowering effect [92,93]. The possible mechanisms of benefit include the inhibition of the sodium-hydrogen exchanger 1 (NHE1) in myocardial cells, leading to reduction in intracellular sodium and calcium concentrations, which was demonstrated on rats and rabbits [94]. Elevated cytosolic levels of these ions might be linked to increased oxidative stress and arrhythmogenesis [95]. Li et al. have demonstrated that SGLT inhibition results in a decrease in collagen type I and type III, and overall cardiac fibrosis in diabetic mice [96]. Also, targeting EAT by SGLT-2 inhibitors might be one of the possible mechanisms of their cardioprotective effect observed in humans. Cinti et al. investigated in their trial the effect of dapagliflozin, a SGLT-2 inhibitor representative, on EAT in patients with T2DM [58]. They showed a rapid 19% reduction in epicardial fat thickness in comparison to baseline values within 4 weeks of treatment. The reduction in EAT thickness was also more pronounced than in other fat depots, potentially due to its high metabolic activity. In the same study, it has been indicated that dapagliflozin reduces the epicardial glucose uptake in patients with T2DM. Glucose uptake was evaluated using positron emission tomography with computed tomography during euglycemic hyperinsulinemic clamp, which provided both metabolic and anatomical information [58]. However, the trial was performed on a small group of patients. Esther Dıaz-Rodrıguez et al. analyzed samples of adipose tissue obtained during cardiac surgeries. In contrast to the previous study, they discovered that incubation of the EAT samples with dapagliflozin showed an increased glucose uptake via glucose transporter 4 (GLUT4) [97]. Requena-Ibáñez et al. conducted a study, which included non-diabetic patients with heart failure with reduced ejection fraction (HFrEF), assessing the effect of empagliflozin on EAT [59]. They reported a significant epicardial fat volume reduction, as well as decrease in interstitial myocardial fibrosis and aortic stiffness. Interstitial myocardial fibrosis was assessed using extracellular volume—a surrogate marker with a prognostic value in HF [98]. SGLT-2 inhibitors have been shown to exert anti-inflammatory effects at both systemic and local levels, which, in turn, reduces cardiovascular risk [60,61,97,99]. For instance, dapagliflozin has been proven to reduce the secretion of inflammatory cytokines, such as C-C motif chemokine ligand 2 (CCL2) in epicardial fat [97]. Dapagliflozin treatment has also been demonstrated to reduce circulating levels of pro-inflammatory TNF-α, which correlated with EAT volume loss [62].

Myasoedova et al., in their meta-analysis comparing the effects of SGLT-2 inhibitors, GLP-1 receptor agonists, and statins on EAT, found GLP-1 receptor agonists to be the most effective in reducing epicardial fat [100]. However, after a 6-month treatment, GLP-1 receptor agonists and SGLT-2 inhibitors were found to have a similar effect. In contrast, Bao et al., in their network meta-analysis, showed SGLT-2 inhibitors’ superiority over GLP-1 agonists or exercise in EAT reduction [101].

### 4.3. Metformin

For many years, the gold standard in the treatment of diabetes mellitus has been metformin [102,103]. It is an oral medication known for its pleiotropic effects. Metformin inhibits liver gluconeogenesis, increases peripheral glucose uptake, and decreases insulin demand, thereby decreasing insulin resistance [104]. Despite its widespread use, the impact of metformin monotherapy on EAT has not been thoroughly investigated. There are only a few studies that have focused on this topic.

Metformin has been demonstrated to significantly influence EAT. Ziyrek et al. showed that metformin monotherapy reduced EAT thickness among newly diagnosed T2DM patients within 3 months of treatment [63]. Similarly, in a study performed by Gunes et al. it has been indicated that metformin treatment significantly reduced EAT thickness in obese children with insulin resistance [64].

EAT plays a key role in developing AF among patients [105,106]. It has been demonstrated that 3 months of metformin monotherapy significantly reduces the atrial electromechanical delay in obese children with insulin resistance [64], a parameter known to predict arrhythmias in studies conducted with both adults and children [107,108,109]. Li et al. have investigated metformin impact on EAT and AF vulnerability on a canine model, in which AF was induced by rapid atrial pacing (RAP) [110]. In this study, metformin reduced RAP-induced EAT and atrial remodeling, up-regulated peroxisome proliferator-activated receptor gamma (PPARγ) expression, and down-regulated pro-inflammatory adipokines expression in the left atrium (LA) and EAT. Metformin modulates activity of enzymes, such as 12-lipoxygenase and AMP-activated protein kinase (AMPK), subsequently enhancing the expression of PPARγ [111,112]. PPARγ shows its anti-inflammatory effect through inhibiting reactive oxygen species (ROS) production, antagonizing NF-κB activation, and promoting adiponectin (APN) release [113]. Increased release of adiponectin leads to anti-inflammatory and anti-oxidation effects, potentially contributing to improvement in atrial remodeling. Additionally, secretion of pro-inflammatory cytokines, such as IL-6 and TNF-α, is significantly reduced by metformin [114,115]. Thus, metformin substantially affects EAT by reducing its thickness and modulating cytokine secretion.

### 4.4. Thiazolidinediones

Thiazolidinediones (TZDs) primarily exert their effects through the activation of PPARγ, a type of nuclear receptor. This impacts particular gene expression and leads to an increase in insulin sensitivity and regulation of lipid metabolism [116]. However, TZDs are not first-line drugs in the treatment of diabetes and are not commonly used due to safety concerns and the availability of newer antidiabetic medications [117,118,119]. TZD therapy is associated with an increase in body weight [120]; therefore, its impact on EAT might be concerning. It turns out that they cause a shift in fat redistribution from visceral adipose tissue (including EAT) to subcutaneous adipose tissue [121]. It has been shown that pioglitazone monotherapy can reduce the epicardial fat thickness [65]. The decrease in the amount of adipose tissue does not apply to pericardial fat. It has been demonstrated that pioglitazone therapy is significantly associated with an increase in pericardial adipose tissue volume, yet this rise did not negatively affect myocardial function under 24 weeks of observation [122]. Additionally, pioglitazone potentially improves the diastolic function among patients with T2DM [65].

In those with coexisting atherosclerosis, pioglitazone has also been found to suppress inflammatory gene expression and attenuate the inflammatory phenotype of EAT [123]. The effect of rosiglitazone, another member of TZDs’ class, on EAT has been evaluated in obese rats with insulin resistance. In this study, it has been demonstrated that rosiglitazone rapidly induces the browning of epicardial fat under 4-day treatment only. It was manifested by a significant shift in the expression of brown fat-specific factors such as UCP-1 or PRDM16. Additionally, rosiglitazone enhances the expression of factors involved in fatty acid oxidation such as g carnitine palmitoyltransferase-1 (CPT1), medium chain acyl-CoA dehydrogenase (MCAD), and very-long chain acyl-CoA dehydrogenase (VLCAD). These molecular changes promote a shift in lipid turnover, contributing to rosiglitazone’s hypolipidemic effect [124]. Despite the beneficial impact of this treatment, it is associated with a higher risk of myocardial infarction [125]. Nevertheless, the number of studies investigating the impact of thiazolidinediones on EAT is limited, and further research is required to comprehensively assess their effects.

### 4.5. Dipeptidyl Peptidase-4 (DPP-4) Inhibitors

Dipeptidyl peptidase-4 (DPP-4) inhibitors are a class of oral medications that influence the incretin system. By inhibiting DPP-4, they prevent the degradation of key incretin hormones, such as GLP-1 and GIP, which increase insulin secretion in a glucose-dependent manner [126]. This results in reduced glucose levels with a low risk of hypoglycemia [127]. The only DPP-4 inhibitor that has been specifically studied so far for its effects on EAT is sitagliptin. It has been demonstrated that sitagliptin causes a rapid reduction in EAT thickness [66]. Interestingly, in this study, the EAT loss did not correlate with changes in BMI and HbA1c%, which might suggest that sitagliptin exerts its effect independently of its beneficial effects on both glucose and lipid profiles. The mechanism behind this remains unknown. Sitagliptin’s effect on epicardial fat has been compared with that of empagliflozin [67]. As far as there were no significant differences between their impact on fat accumulation, cardiac function, and cardiac fatty acid metabolism, empagliflozin significantly decreased serum uric acid and increased HDL cholesterol, ketone bodies, and insulin sensitivity when compared to the values observed with sitagliptin. Therefore, the usage of empagliflozin might be favored over prescribing sitagliptin.

Hirose et al., in their study, have demonstrated that DPP-4 inhibition may result in a decrease in the myocardial fibrosis by inhibiting the production of collagen type III in mice after transverse aortic constriction (TAC) [128]. In contrast, Mulvihill et al. have demonstrated that high-fat fed diabetic mice treated with MK-0626, a highly selective DPP4 inhibitor, presented exacerbated cardiac fibrosis, inflammation, and also impaired ventricular function [129]. These studies do not analyze the role of EAT; however, there is a possibility that EAT may mediate the effects of DPP-4 inhibitors on the cardiac muscle.

### 4.6. Insulin

Data regarding the impact of insulin treatment on EAT are limited. It is known that insulin stimulates lipogenesis and lipid storage in adipose tissue [130,131]. Akt signaling activated by the stimulation of an insulin receptor (INSR) is essential for adipogenesis [132,133]. One of its effectors is PPARγ, the transcriptional regulator of adipocyte differentiation [134]. Trabzon et al., in their study, demonstrated that children with type 1 diabetes presented with significantly higher EAT thickness than healthy individuals, suggesting that the increase in the amount of EAT may be related to the insulin dose used in treatment [135]. Surprisingly, in a pilot study performed by Elisha et al., it has been shown that initiation of both insulin analogs—Detemir and Glargine—among insulin-naïve and inadequately controlled patients with T2DM led to a reduction in EAT thickness [68]. The decrease was more pronounced in patients receiving Detemir.

### 4.7. Sulfonylureas

No studies regarding the influence of sulfonylureas on EAT were found.

## 5. Non-Pharmacological Interventions

**Figure 1:**
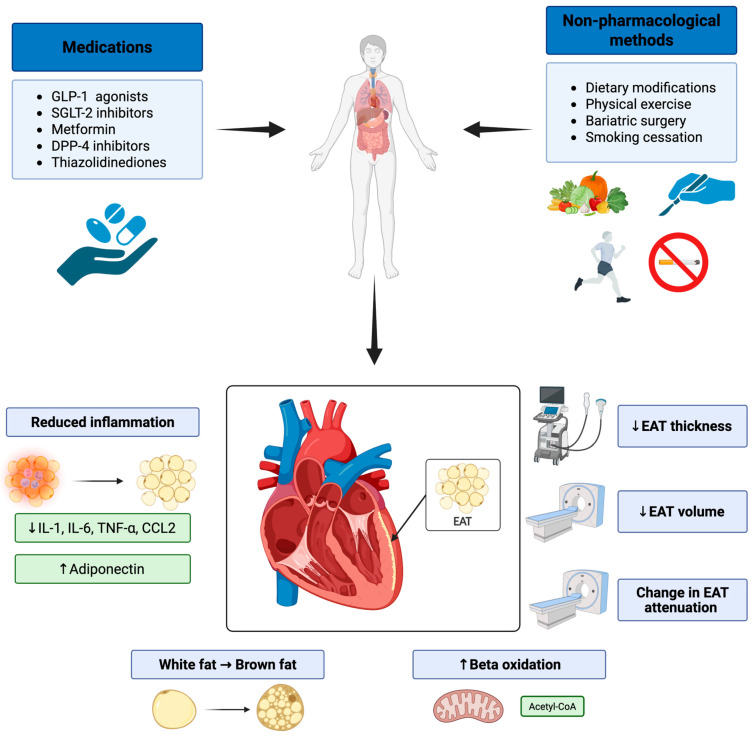
Possible impact of pharmacological and non-pharmacological diabetes mellitus treatment options on epicardial adipose tissues with potential mechanisms. Abbreviations: CCL2, C-C Motif Chemokine Ligand 2; DPP-4, Dipeptidyl Peptidase 4; EAT, Epicardial Adipose Tissue; GLP-1, Glucagon Like Peptide 1; IL-1, Interleukin 1; IL-6, Interleukin 6; SGLT-2, sodium-glucose cotransporter-2; TNF-α, Tumor Necrotic Factor Alpha. Symbols: ↓, a decrease; ↑, an increase. Created in BioRender. Krauz, K. (2025) https://BioRender.com/hunvhri (accessed on 14 July 2025).

Even though medical therapy is used for DM treatment, non-pharmacological approaches are necessary in the management of DM (Figure 1). Nutritional and lifestyle changes must be advised to patients. Some individuals may require bariatric surgery to alleviate coexisting obesity [136]. The evidence regarding the impact of those interventions on EAT is limited, especially directly in the population of patients with DM. However, existing data are summarized below. 

Generally, EAT is affected by weight fluctuations. Nakazato and colleagues analyzed the relationship between EAT volume and alterations in body weight. The data were retrospectively assessed from 374 patients from the EISNER registry, who underwent two CT scans, with a mean interim of 4.1 ± 0.4 years between the studies. Change in EAT volume was positively correlated with weight change, BMI change, and waist circumference change [137].

### 5.1. Physical Exercise

Training is a non-pharmacological approach to lowering blood glucose levels and controlling diabetes. Moderate-to-high-intensity exercise should be implemented in T2DM patients with a minimal duration of 150 min per week. To sustain its benefits, the patients should avoid breaks longer than two consecutive days, which underlines the necessity of being systematic [136]. There was an inverse association between physical activity and DM incident, suggesting that it may also play a part in the disease [138]. Data from the included clinical studies are summarized in Table 2.

Honkala et al. analyzed the impact of a 2-week period (six sessions) of high-intensity interval training and moderate-intensity continuous training on EAT in healthy individuals and those with defective glucose tolerance (DGT). Before the intervention, EAT volume was significantly higher in the DGT group than in the healthy group. Both types of training significantly reduced EAT volume in the healthy and DGT populations [139]. In another study performed by Jonker and colleagues, 12 patients with T2DM underwent a 6-month individualized training program followed by a 12-day trekking expedition at a high altitude. Before and after the intervention, the participants had MRI scans to assess visceral and subcutaneous fat. The EAT volume did not change after training. No changes were also observed in the subcutaneous fat and cardiac function parameters assessed in transthoracic echocardiography. However, visceral abdominal fat volume was significantly reduced [140]. These results may suggest that different fat depots respond differently to physical exercise.

Most data come from studies whose population did not primarily involve only DM patients. The results of a pilot study revealed that 3-week high-intensity, moderate-volume muscular-endurance resistance training significantly reduced EAT volume in obese females compared to non-exercising controls [141]. Similarly, Christensen et al., in the secondary analysis of a randomized clinical trial, investigated whether 12-week endurance or resistance training impacts EAT mass in physically inactive patients with abdominal obesity. The results demonstrated that both types of activity significantly reduced EAT mass, determined from MRI scans, whereas no difference was found in the control group [142]. In another pilot study, patients on maintenance hemodialysis received either a 4-month intradialytic endurance exercise training program consisting of cycling 3 days per week or standard care. EAT thickness was significantly reduced after 4 months in the exercising group. The change in EAT thickness inversely correlated with changes in physical performance assessed by the shuttle walk test [143]. Rosety et al. performed a randomized controlled study and analyzed the impact of a 12-week resistance circuit training program (3 days per week) on EAT thickness assessed in transthoracic echocardiography in obese aged females. In the experimental group, EAT thickness was significantly reduced after the resistance training program [144]. Aerobic exercise training performed by obese middle-aged men 3 days a week for 12 consecutive weeks resulted in a decrease in EAT thickness assessed by transthoracic echocardiography. Moreover, the percentage change in EAT thickness was more pronounced than changes in BMI or body weight. The change in EAT thickness was also associated with a decrease in visceral adipose tissue [145].

Exercise snacks are short and intensive training sessions lasting maximally 1 min and are repeated throughout the day [147]. Zhou and colleagues randomly subjected participants to the snacks group, which performed 4 days of sprint exercises, by stair-climbing, per week for 12 consecutive weeks. The control group consisted of those who were excluded from any exercises. After this period, a significant decrease in EAT volume, assessed by CT, was demonstrated in the snack group. This change was accompanied by a decrease in total fat mass. Moreover, after 12 weeks, the EAT volume of the snacks group was significantly lower than in the control group, even though no difference occurred at the beginning [146].

### 5.2. Diet

Maintaining a proper diet in DM patients is crucial not only for blood glucose control but also for management of other cardiovascular risk factors like hypertension, obesity, and hyperlipidemia [148]. Data from included clinical studies are summarized in Table 3.

Severely obese individuals (n = 20) were subjected to a weight loss program including a very low-calorie diet. After 6 months, BMI and EAT thickness significantly decreased. These changes were accompanied by a decrease in left ventricular mass and an increase in E/A ratio and isovolumic relaxation time [149]. Similarly, obese men followed a 12-week study including a calorie-restricted diet. After this period, the average decrease in epicardial fat thickness was 17.2%. This change was related to the decrease in visceral adipose tissue loss [150]. Also, Barrio-Lopez et al. demonstrated that, among patients undergoing atrial fibrillation ablation, adherence to the Mediterranean diet was inversely related to average EAT [152]. Moreover, supplementation of docosahexaenoic acid for 6 months among overweight children with nonalcoholic fatty liver disease resulted in a significant reduction in EAT thickness [151]. Among middle-aged individuals, EAT thickness was positively associated with red meat intake and negatively associated with fruit consumption. In contrast, in women, it was directly related to alcohol intake and heavy drinking [153].

Data from studies on animal models provide evidence that diet may impact not only the amount of EAT but also its composition and, therefore, it probably affects myocardial function. Feeding male C57Bl/6J mice with a high-fat diet for 12 weeks increased their body weight. No changes were observed in the levels of fasting blood glucose. However, serum insulin levels were significantly raised. High-fat diet promoted an increase in epicardial + pericardial adipose tissues and contributed to greater expression of TNF-α, MCP-1, and TGF-β1 and lower expression of adiponectin in those tissues, suggesting a shift towards pro-inflammatory and pro-fibrotic profile [154]. A study on Ossabaw miniature pigs revealed that diet affects the composition of EAT. Animals fed with a diet rich in unsaturated fat had higher content of PUFAs in EAT, which were linked with expression of genes linked with anti-inflammatory signaling. These results may be extrapolated to humans, indicating that a diet rich in unsaturated fat may reduce inflammation in EAT. In contrast, pigs on the Western diet, rich in saturated fat, had a greater proportion of total SFAs and trans fatty acids, which was related to pro-inflammatory gene expression [155]. Similarly, Ossabaw pigs on a Western diet had upregulated the radical S-adenosyl methionine domain-containing protein two gene in EAT, which is induced by interferon signaling, indicating activation of immune response mediated by interferon [156]. New Zealand white rabbits, fed with a diet containing an increased amount of cholesterol, had significantly higher content of 16:0 (Palmitic acid) and 18:1t (Oleic acid) fatty acids, and decreased content of 18:1 n-9 (Oleic acid), 18:2 n-9 (Linoleic acid), and 18:3 n-9 (Linolenic acid) in EAT [157]. These data suggest that diet may affect lipid composition in EAT. In another study, domestic male swine were fed high-calorie diets. The animals had significantly higher EAT thickness than the control group. Moreover, animals treated with a high caloric diet and ezetimibe had significantly reduced EAT thickness compared to the group with a high caloric diet only [158].

### 5.3. Bariatric Surgery

Bariatric surgery is an effective treatment method for morbid obesity and T2DM to date, which usually improves related metabolic problems [159,160]. Different methods of bariatric surgery are described, and they can differ in the efficacy of weight loss following the procedure. A substantial number of studies examined the effects of bariatric surgery and the following weight loss on EAT thickness or volume at different time points. The data are summarized in Table 4.

Hunt and colleagues found that patients with a history of bariatric surgery had significantly lower EAT volume than obese individuals who were not operated on [172]. Ten obese patients with insulin-dependent T2DM underwent Roux-en-Y gastric bypass surgery (RYGB). There was a significant decrease in EAT volume assessed 16 weeks after surgery. No changes in myocardial triglyceride content were found [161]. Similarly, another study on this matter investigated the impact of bariatric surgery on cardiac ectopic fat, where MRI was used to measure EAT volume in 23 morbidly obese patients before the surgery, and 6 months after the procedure. Mean EAT volume was significantly reduced after the surgery. While epicardial fat values changed significantly, myocardial triglyceride content remained unchanged. Interestingly, patients with sleep apnea had a smaller reduction in EAT volume [162].

In a study by Meulendijks et al., 37 obese individuals underwent CT scans and serum collection before and after bariatric surgery. Serum collection was performed to examine pro- and anti-inflammatory adipokines. After the surgery, on follow-up, there was a significant decrease in EAT volume and an increase in EAT attenuation. A higher serum adiponectin was associated with a lower EAT volume and higher EAT attenuation [163]. Willens et al. evaluated the impact of weight loss after bariatric surgery on EAT thickness changes. A total of 23 patients were followed up 8.3 ± 3.7 months after the surgery. Their EAT thickness was significantly lower than at the baseline. EAT thickness at the beginning was a predictor of its change [164].

Graziani et al. assessed the impact of bariatric surgery on cardiac remodeling. In a follow-up, two years after the surgery, EAT thickness was significantly lower in comparison to the baseline. EAT thickness also positively correlated with BMI, left ventricular end diastolic volume (a parameter which increases in volume overload), left ventricular end systolic volume (which is increased when contractility is impaired), and LV mass, and negatively correlated with systolic function expressed as LVEF [165].

Altin and colleagues analyzed the influence of weight loss after laparoscopic sleeve gastrectomy on EAT thickness and carotid intima media thickness (CIMT). A total of 6 months following the procedure, the values of both variables were significantly lower than at the beginning of the study. There was a significant positive correlation between the CIMT change and the change in EAT thickness [166]. Analysis of the impact of weight loss after laparoscopic sleeve gastrectomy on markers of atherosclerotic vascular disease showed that at the 6-month follow-up, EAT thickness significantly decreased. These changes significantly correlated with the change in CIMT and aortic propagation velocity, which may be used in assessing CAD burden [167].

Kokkinos et al. compared the effects of two types of bariatric surgery, RYGB and sleeve gastrectomy, on cardiovascular indices. There were no differences in terms of BMI before as well as 3 and 6 months following the surgery. At 6 months after the surgery, the EAT thickness values were significantly lower in both groups compared to the baseline. At follow-up, the EAT thickness of patients who underwent gastric bypass surgery was significantly lower than in those who underwent sleeve gastrectomy, even though there was no difference between the groups in baseline [168]. Henry and colleagues compared the effects of different bariatric surgery types: RYGB, laparoscopic sleeve gastrectomy (LSG), and Laparoscopic Adjustable Gastric Band (LAGB) on cardiac reverse remodeling. The decrease in EAT was more significant in the RYGB and LSG groups compared to the LAGB group. There was a positive correlation between changes in EAT and changes in the left ventricular eccentricity index, which is a marker of pericardial restraint [169]. In another study, Henry et al. reported that a substantial reduction in EAT was observed in the first 212 days following surgery [170]. In contrast, Asteria et al. did not find a difference in EAT volume assessed 12 months following surgery between patients undergoing LSG and RYGB. However, the EAT volume was reduced compared to the baseline [171].

### 5.4. Combination or Comparison of Weight Reduction Methods in Terms of EAT Changes

Several studies assessed the impact of both dietary intervention and physical exercise on EAT. Leroux-Stewart et al., in a pilot study, analyzed the impact of caloric restriction combined with physical activity for 16 weeks on EAT thickness and total fat mass among T2DM individuals. The addition of physical activity to the caloric deficit leads to a more pronounced reduction in both total fat mass and EAT thickness. However, these changes were not accompanied by changes in arterial pressure, HbA1c, and lipid profile [173].

We et al. compared the impact of bariatric surgery and weight loss induced by exercise and diet on visceral fat depots. After 3 months from baseline, the EAT volume significantly decreased in both groups. EAT volume change in the bariatric surgery group was significantly greater than in the other group. A similar relation was observed for BMI changes. The changes in EAT volume in both groups were significantly lower than the changes in other analyzed fat depots—abdominal subcutaneous adipose tissue, abdominal visceral adipose tissue, and paracardial adipose tissue [174].

Serrano-Ferrer et al. analyzed the impact of a 6-month lifestyle intervention on regional myocardial function in patients with metabolic syndrome. A total of 87 patients were randomized into groups with different resistance and endurance for the training, and received a standardized nutritional intervention, including caloric deficit. At follow-up, the EAT thickness significantly decreased in comparison to the baseline. Moreover, changes in EAT thickness were independent predictors of LV longitudinal strain changes [175]. Similarly, 188 severely obese individuals (BMI 40.2 ± 8.6 kg/m^2^) were assigned to a 1-year weight reduction program, which included dietary and lifestyle intervention. This group comprised 71 patients with LVDD. Among patients with successful weight reduction, EAT thickness significantly decreased compared to the baseline value. EAT thickness reduction also predicted the improvement of diastolic function, as large EAT volume may mechanically impair diastolic filling [176]. Another study included 74 obese women who were subjected to a 1-year weight reduction program, which included dietary and exercise intervention and cognitive behavioral therapy, if necessary. A total of 28 women completed the program. Among them, patients with a reversal of metabolic syndrome had a significantly greater reduction in EAT thickness than subjects whose status of metabolic syndrome did not change [177]. These data support the hypothesis that changes in EAT are a reflection of systemic modifications and, as a local mediator, EAT may convey these changes to the heart. In another study, overweight or mildly obese persons were randomly assigned to receive a 12-week aerobic exercise program, or to the control group. Both groups were instructed about a healthy weight maintenance diet, but no calorie restriction was applied. The exercise program significantly reduced EAT thickness. This change was accompanied by a weight reduction [178]. Also, Liang and colleagues analyzed the impact of a 3-month weight reduction program on the relationship between insulin resistance and EAT thickness. The study included 32 non-diabetic, obese men with metabolic syndrome. The weight loss program comprised dietary intervention and physical exercise. EAT thickness in all studied regions significantly decreased following the weight loss program. The change in HOMA-IR index (homeostasis model assessment index of insulin resistance), calculated as fasting glucose mg/dL × fasting insulin μU/mL/405, positively correlated with the decrement ratio of EAT thickness in the superior interventricular groove [179]. Changes in right atrioventricular groove EAT thickness also significantly correlated with changes in soluble CD40 ligand, which may induce pro-inflammatory effects [180].

There are also conflicting results regarding this topic. In another study, ten obese patients were assigned to undergo a weight reduction program including a low-calorie diet and aerobic exercise. The mean weight loss duration was 44.1 ± 27.6 days. After this period, no differences were observed in the EAT volume, even though the volume of both visceral and subcutaneous adipose tissue significantly decreased, which supports the hypothesis that EAT volume is more resistant to changes than other fat depots [181].

### 5.5. Smoking Cessation

Smoking is a known risk factor for numerous cardiovascular diseases. Smoking patients not only have a higher chance of developing DM, but it also exacerbates the progression of the disease [182].

A trial by Mach et al. showed significantly increased TNF-α and IL-6 tissue concentration levels in EAT samples of current smokers in comparison to former smokers and never smokers. Chronic inflammation, reflected by an increase in expression levels of TNF-α and IL-6, was presented as a risk factor for developing insulin resistance and T2DM [183]. Moreover, TNF-α reduces expression of GLUT-4, an insulin-dependent glucose transporter that enables cells to absorb glucose, which promotes insulin resistance [184]. Comparison of former smokers (patients who smoked more than 100 cigarettes in their life but were not currently smoking) and never smokers (patients that did not smoke more than 100 cigarettes in their life) showed no significant difference in cytokine production, which marks the importance of smoking cessation [185].

Milanese et al. assessed the correlation between EAT features and morphometric, demographic, and clinical data in 1379 patients. The results showed that smoking was associated with a higher EAT volume [186]. Hartiala et al. quantified EAT volume in 557 Finnish patients, aged 40–46 years. Smoking habit data were gathered through a questionnaire. In a multivariable regression model, the history of smoking was positively correlated with EAT volume [187]. Similarly, Foldyna et al. extracted data from CT scans of 24,090 individuals who were current/recent or former heavy smokers (>30 pack-years) aged between 55 and 74 years. EAT volume and density values were measured using deep machine learning algorithms. The value used in all analyses was Body Surface Area (BSA) indexed mean EAT volume. The results revealed that EAT volume was significantly higher in the former than in current smokers. Moreover, EAT volume positively correlated with pack years, and EAT density was negatively correlated with smoking habits [188]. Another study by Monti et al. focused on patients with metabolic syndrome (MetS) and aimed to assess the correlation between EAT and smoking. A total of 54 adult individuals between 45 and 75 years old and diagnosed with MetS underwent cardiac CT scans and were divided into two groups depending on their smoking habits. Inclusion criteria for the smoking group were smoking >10 cigarettes a day for the last 10 years. Non-smokers consisted of patients who either did not smoke cigarettes or had smoked less than 100 cigarettes in their lifetime. The smokers group had markedly higher EAT volume independent of age. Additionally, smoking was the best variable in predicting epicardial fat [189].

A view from a different angle was implemented by Gać et al. [190], who focused on tobacco smoke exposure (ETS) and EAT thickness specifically in hypertensive patients. The Secondhand Smoke Exposure Scale (SHSES) was used to divide 96 patients into groups. EAT thickness was measured using CT. Patients exposed to tobacco smoke at home daily had significantly higher EAT thickness values than the patients who were not exposed at home daily. This was also the case for patients exposed to tobacco smoke in the car daily and for the group that experienced ETS in public places in the last week. Interestingly, no significant difference has been established between patients who experienced ETS at work for the past week and those who were not exposed to ETS at work for the past week. A positive correlation between EAT thickness and SHSES points was revealed in groups with both mild and moderate hypertension [190].

The studies listed above reinforce the conclusion that smoking may change the phenotype of EAT by promoting an increase in its volume and thickness and lowering its attenuation. Additionally, it impacts the transcriptome of EAT and exacerbates inflammatory signaling. Taken together, these data highlight the importance of smoking cessation in managing cardiometabolic risk.

## 6. Conclusions and Future Directions

Changes in EAT may be a reflection of systemic metabolic disorders and, therefore, they may exert impact on the myocardium, serving as a potential mediator. Multiple studies indicated that both pharmacological and non-pharmacological DM treatment methods may affect EAT. The question arises whether this effect is direct or whether the reduction in blood glucose levels and the modulation of other cardiovascular risk factors indirectly influence EAT parameters. Research investigating the effect of anti-diabetic drugs on EAT has yielded promising results, suggesting potential cardiovascular benefits. Those include EAT reduction, and local and systemic anti-inflammatory influence. The majority of the presented data concerning non-pharmacological approaches come from studies that do not involve patients with DM exclusively. Also, little is known about the effects of non-pharmacological methods on EAT composition, as studies rather describe changes assessed by imaging studies. However, such studies on humans could raise ethical concerns as they would require an EAT biopsy. Due to this fact, studies examining the impact of anti-diabetic treatment on molecular mechanisms in EAT are required. As novel medications, like TZT, emerge, it would be interesting to conduct randomized controlled trials assessing the correlation between EAT parameter changes and cardiovascular event rates following TZT therapy. Also, nutraceuticals and supplements may be considered useful in management of diabetes or prediabetes [191]. However, little is known about the impact of such compounds on quantity and quality of EAT, and this warrants further attention.

The possibility of including EAT parameters as a novel element in cardiovascular risk assessment scores would be an innovative approach in the future. Even though the evidence is promising, several problems emerge and need to be overcome. Firstly, previous studies use different ranges of EAT attenuation for EAT quantification in CT imaging. Also, variability in CT acquisition protocols may occur between centers including, for example, distinct tube voltages. These factors may restrict the comparability of results. Further studies are required to standardize the details of EAT examination. Moreover, implementation of AI-driven tools and machine learning may help improve reproducibility and cost-effectiveness of EAT examination.

## Figures and Tables

**Table 1:** Summary of clinical studies on the effect of pharmacological interventions on EAT.

Author, Reference	Pharmacological Intervention	Study Population	Method of EAT Assessment	Observation Time	Change in EAT Characteristics
**GLP-1 receptor agonists**					
Morano et al. [54]	Exenatide or liraglutide	25 patients with T2DM exenatide: *n = 12* liraglutide: *n = 13*	Echo	3 months	↓ EAT thickness in both exenatide and liraglutide groups No significant difference between groups
Iacobellis et al. [55]	Metformin or metformin + liraglutide	95 patients with T2DM metformin + liraglutide: *n = 54* metformin: *n = 41*	Echo	3, 6 months	↓ EAT thickness in metformin + liraglutide group No significant difference in metformin group
Iacobellis et al. [56]	Semaglutide or dulaglutide or metformin	80 patients with T2DM and obesity Addition of semaglutide to the current diabetes treatment: *n = 30* Addition of dulaglutide to the current diabetes treatment: *n = 30* Newly diagnosed patients who were started on metformin and received dietary recommendations: *n = 20*	Echo	12 weeks	↓ EAT thickness in semaglutide and dulaglutide groups, no significant difference between them No significant EAT thickness reduction in metformin group
Kramer et al. [57]	Tirzepatide	175 patients with obesity-related HFpEF	MRI	52 weeks	No significant changes in EAT volume
**SGLT-2 inhibitors**					
Cinti et al. [58]	Dapagliflozin	14 patients with T2DM dapagliflozin: *n = 7* placebo: *n = 7*	PET/CT during Euglycemic hyperinsulinemic clamp	4 weeks	↓ EAT thickness ↓ glucose uptake by EAT
Requena-Ibáñez et al. [59]	Empagliflozin	84 nondiabetic HFrEF patients empagliflozin: *n = 42* placebo: *n = 42*	MRI	6 months	↓ EAT volume
Fukuda et al. [60]	Ipragliflozin	9 non-obese patients with T2DM	MRI	12 weeks	↓ EAT volume,
Bouchi et al. [61]	Luseogliflozin	19 patients with T2DM	MRI	12 weeks	↓ EAT volume
Sato et al. [62]	Dapagliflozin	40 patients with T2DM and CAD dapagliflozin: *n = 20* conventional treatment: *n = 20*	CT	6 months	↓ EAT volume
**Metformin**					
Ziyrek et al. [63]	Metformin	40 patients newly diagnosed with T2DM	Echo	3 months	↓ EAT thickness
Gunes et al. [64]	Metformin	30 obese children with insulin resistance	Echo	3 months	↓ EAT thickness
**Thiazolidinediones**					
Moody et al. [65]	Pioglitazone	24 individuals 12 patients without CVD 12 subjects with normal glucose tolerance	MRI	24 weeks	↓ EAT volume
**DPP-4 inhibitors**					
Lima-Martinez et al. [66]	Sitagliptin	26 patients with T2DM inadequately controlled with metformin	Echo	24 weeks	↓ EAT thickness
Hiruma et al. [67]	Sitagliptin or empagliflozin	44 patients with T2DM sitagliptin: *n = 23* empagliflozin: *n = 21*	MRI	12 weeks	↓ EAT area No significant difference in EAT reduction between groups
**Insulin**					
Elisha et al. [68]	Insulin Detemir or Insulin Glargine	36 patients inadequetly controlled T2DM Insulin Glargine: *n = 20* Insulin Detemir: *n = 16*	Echo	6 months	↓ EAT thickness No significant difference between groups

Abbreviations: CAD, coronary artery disease; CT, computed tomography; CVD, cardiovascular disease; Echo, echocardiography; HFpEF, heart failure with preserved ejection fraction; HFrEF, heart failure with reduced ejection fraction; MRI, magnetic resonance imaging; PET/CT, positron emission tomography/computed tomography; T2DM, type 2 diabetes mellitus. ↓ indicates a decrease.

**Table 2:** Summary of the described effects of physical exercise on EAT characteristics.

**Author,** **Reference**	**Intervention**	**Study Population**	**Method of EAT Assessment**	**Observation Time**	**Change in EAT Characteristics**
Honkala et al. [139]	HIIT or MICT	44 men healthy: *n = 28* DGT: *n = 16*	CT	2 weeks	↓ EAT volume
Jonker et al. [140]	moderate-intensity exercise followed by a high-altitude trekking expedition with exercise of long duration	12 patients with T2DM	MRI	6 months	No significant changes in EAT volume
Fernandez-del-Valle et al. [141]	high-intensity, moderate-volume muscular endurance resistance training	11 young females with obesity * intervention: *n = 6* control: *n = 5*	MRI	3 weeks	↓ EAT volume in intervention group
Christensen et al. [142]	endurance or resistance training	39 physically inactive participants with abdominal obesity ^a^ endurance: *n = 14* resistance: *n = 13* control: *n = 12*	MRI	12 weeks	Decrease in EAT mass significantly greater in endurance and resistance groups compared to the control group.
Wilund et al. [143]	intradialytic exercise training (cycling)	17 hemodialysis patients exercising: *n = 8* (43% diabetic) control group: *n = 9* (50% diabetic)	Echo	4 months	↓ EAT thickness in the exercising group
Rosety et al. [144]	circuit training program, 3 days per week	48 obese women over 65 years old ^a^ intervention: *n = 24* control: *n = 24*	Echo	12 weeks	↓ EAT thickness in the exercising group
Kim et al. [145]	supervised exercise training program	24 obese middle-aged men (57.7% with metabolic syndrome)	Echo	12 weeks	↓ EAT thickness
Zhou et al. [146]	4 days of sprint exercises by stair-climbing per week (“exercise snacks”)	27 participants * snack group: *n = 14* control group: *n = 13*	CT	12 weeks	↓ EAT volume

* No data regarding the number of patients with carbohydrate metabolism disorders or diabetes. ^a^ Diabetes was an exclusion criterion. Abbreviations: CT, computed tomography; DGT, defective glucose tolerance; EAT, epicardial adipose tissue; Echo, echocardiography; HIIT, high-intensity interval training; MICT, moderate-intensity continuous training; MRI, magnetic resonance imaging; T2DM, type 2 diabetes mellitus. ↓ indicates a decrease.

**Table 3:** Summary of data from clinical studies about the effects of diet on EAT characteristics.

Author, Reference	Intervention	Study Population	Method of EAT Assessment	Observation Time	Change in EAT Characteristics
Iacobellis et al. [149]	very low-calorie diet weight loss program	20 severely obese individuals ^a^	Echo	6 months	↓ EAT thickness
Kim et al. [150]	low-calorie diet	27 moderately obese men *	Echo	12 weeks	↓ EAT thickness
Pacifico et al. [151]	DHA supplementation	51 overweight children ^a^ DHA: *n = 25* placebo: *n = 26*	Echo	6 months	↓ EAT thickness

* No data regarding the number of patients with carbohydrate metabolism disorders or diabetes. ^a^ Diabetes was an exclusion criterion. Abbreviations: EAT, epicardial adipose tissue; Echo, echocardiography; and DHA, docosahexaenoic acid. ↓ indicates a decrease.

**Table 4:** Summary of the described effects of bariatric surgery on EAT characteristics.

Author, Reference	Intervention	Study Population	Method of EAT Assessment	Observation Time	Change in EAT Characteristics
van Schinkel et al. [161]	RYGB	10 obese patients with T2DM	MRI	16 weeks	↓ EAT volume
Gaborit et al. [162]	RYGB	23 morbidly obese patients (26% DM)	MRI	6 months	↓ EAT volume
Meulendijks et al. [163]	RYGB or SG	36 patients over 40 years old (5% DM)	CT	1 year	↓ EAT volume ↑ EAT attenuation
Willens et al. [164]	open or laparoscopic RYGB laparoscopic vertical band gastroplasty LAGB	23 patients with severe obesity *	Echo	8.3 ± 3.7 months	↓ EAT thickness
Graziani et al. [165]	bariatric surgery (not specified)	80 obese patients * surgery: *n = 51* controls: *n = 29*	Echo	2 years	↓ EAT thickness
Altin et al. [166]	laparoscopic SG	105 patients (26.7% DM)	Echo	6 months	↓ EAT thickness
Kaya and Elkan [167]	laparoscopic SG	71 patients (36.6% DM)	Echo	6 months	↓ EAT thickness
Kokkinos et al. [168]	RYGB or SG	37 patients RYGB: *n = 14* (21.4% DM) SG: *n = 23* (26.1% DM)	Echo	6 months	↓ EAT thickness in both groups; significantly greater decrease in RYGB group
Henry et al. [169]	RYGB or laparoscopic SG or LAGB	58 patients RYGB: *n = 26* (12% T2DM) laparoscopic SG: *n = 22* (18% T2DM) LAGB: *n = 10* (0% T2DM)	MRI	short-term (median 251–273 days) and longer-term (median 983–1027 days)	↓ EAT volume; significantly greater decrease in RYGB group
Henry et al. [170]	gastric bypass or SG or adjustable gastric band	62 patients (15% diabetes) gastric bypass: *n = 28*; SG: *n = 26*; adjustable gastric band: *n = 8*	MRI	short-term (median 212 days), medium-term (median 428 days), long-term (median 1030 days)	↓ EAT volume
Asteria et al. [171]	laparoscopic SG or RYGB	15 patients * RYGB: *n = 6* SG: *n = 9*	MRI	12 months	↓ EAT volume; significantly greater decrease in RYGB group

* No data regarding the number of patients with carbohydrate metabolism disorders or diabetes. Abbreviations: CT, computed tomography; EAT, epicardial adipose tissue; Echo, echocardiography; LAGB, Laparoscopic Adjustable Gastric Band; MRI, magnetic resonance imaging; RYGB, Roux-en-Y Gastric Bypass; SG, sleeve gastrectomy; and T2DM, type 2 diabetes mellitus. Symbols: ↓, a decrease; ↑, an increase.

## Data Availability

Not applicable. No new data were created.
